# Interactions of genetic variants and prenatal stress in relation to the risk for recurrent respiratory infections in children

**DOI:** 10.1038/s41598-021-87211-0

**Published:** 2021-04-07

**Authors:** Laura S. Korhonen, Minna Lukkarinen, Katri Kantojärvi, Panu Räty, Hasse Karlsson, Tiina Paunio, Ville Peltola, Linnea Karlsson

**Affiliations:** 1 Department of Clinical Medicine, FinnBrain Birth Cohort Study, Turku Brain and Mind Center, University of Turku, Turku, Finland; 2 Department of Paediatrics and Adolescent Medicine, Turku University Hospital and University of Turku, P.O. Box 52, 20521 Turku, Finland; 3 Genomics and Biobank Unit, Finnish Institute for Health and Welfare, Helsinki, Finland; 4 Department of Psychiatry and SleepWell Research Program, Faculty of Medicine, University of Helsinki and Helsinki University Central Hospital, Helsinki, Finland; 5 Department of Psychiatry, Turku University Hospital and University of Turku, Turku, Finland; 6 Centre for Population Health Research, University of Turku and Turku University Hospital, Turku, Finland

**Keywords:** Developmental biology, Genetics, Immunology, Medical research, Risk factors

## Abstract

Genetic variants may predispose children to recurrent respiratory infections (RRIs) but studies on genotype-environment interaction are rare. We hypothesized that the risk for RRIs is elevated in children with innate immune gene variants, and that prenatal exposure to maternal psychological distress further increases the risk. In a birth cohort, children with RRIs (n = 96) were identified by the age of 24 months and compared with the remaining cohort children (n = 894). The risk for RRIs in children with preselected genetic variants and the interaction between maternal distress during pregnancy and child genotype were assessed with logistic regression. The *IL6* minor allele G was associated with elevated risk for RRIs (OR 1.55; 95% CI 1.14–2.12). Overall, there was no interaction between maternal psychological distress and child genotype. Exploratory analyses showed that, the association between the variant type of *IL6* and the risk for RRIs was dependent on prenatal exposure to maternal psychological distress in males (OR 1.96; 95% CI 1.04–3.67). Our study didn’t find genotype-environment interaction between prenatal maternal distress and child genotype. Exploratory analyses suggest sex differences in gene-environment interaction related to susceptibility to RRIs.

## Introduction

Respiratory tract infections (RTIs) account for the major part of infectious diseases among infants and children, causing a substantial disease burden for individuals and a financial burden for the society^1^. RTIs are mostly caused by respiratory viruses circulating in the community but they are often complicated by acute otitis media (AOM) and antibiotic use is common^2^. It has been reported that 10% of children under 2 years of age suffer from recurrent upper and lower RTIs^3^. These children, regarded as having recurrent respiratory infections (RRIs), use much more health care services and antibiotics than other children, and they often undergo surgical procedures, such as tympanostomy tube insertion or adenoidectomy.

Reasons why children under 2 years of age are susceptible to respiratory tract infections include the declining levels of maternal antibodies derived through placenta and child’s own immature adaptive immune responses^4^. Therefore, innate immunity is particularly important in response to infectious agents in infants and toddlers. However, there are factors impairing the functions of innate immune system and thereby increasing susceptibility to infections. Common genetic variants of pattern recognition receptor family toll-like receptors (TLR)^5^, mannose-binding lectin (MBL)^6,7^, and cytokines play a role in immunocompetence^8^. Polymorphisms of *TNF, IL6,* and *IL10* genes have been reported to specifically affect the susceptibility to RTIs^8–11^. Environmental risk factors, such as parental smoking during and after pregnancy^12,13^, lower socioeconomic status of the family^14^, shorter duration of breastfeeding^2,13^, (greater) number of siblings^13^, male sex^15^, outside-home daycare^13^, and, as recently recognized, maternal psychological distress during pregnancy have been identified to increase the susceptibility to RTIs in children^16,17^. The genotype–environment interaction (GxE), i.e. the dependence of individual’s susceptibility to environment risk factors on presence of certain single nucleotide polymorphisms (SNPs) or other genetic variations has recently gathered interest. To our knowledge, there are no studies on the interaction of genetic variants of innate immunity and maternal prenatal psychological distress related to RTI, although it has been previously established that they both alone increase the risk for RRIs.

In this study, we first aimed to analyze how common genetic variants reportedly affecting the function of innate immune system (in genes *IL6, IFI44L, IL10, IFIH1, MBL2, IL17A, TLR4*, *TLR2*, *IL4,* and *TNF*) associate with RRIs in young children. Secondly, since in our previous study prenatal maternal psychological distress was linked to elevated risk for child’s RRIs^16^, we aimed to test the genotype–environment hypothesis by exploring the interactions of prenatal exposure to maternal psychological distress and genetic variants of innate immunity in relation to the risk for RRIs in the child. Thirdly, as females and males differ in their innate immune and stress responses, we aimed to explore the difference separately in both sexes.

## Methods

### Study cohort and data sources

This study was conducted within prospective observational FinnBrain Birth Cohort Study (www.finnbrain.fi)^18^. The baseline recruitment for the Cohort took place at gestational weeks (gwks) 12 in Turku and the Åland Islands, Finland between December 2011 and April 2015. The Cohort consisted of 3808 women and their 3837 children Mothers were considered eligible to participate in the study if they had a verified pregnancy and sufficient knowledge of Finnish or Swedish to fill in the study questionnaires. The research questionnaires were either mailed to participants or could be completed online at the discretion of each participant. The parents answered to the questionnaires at gwks 14, 24, and 34 as well as at child ages of 3, 6, 12, and 24 months. The data on background factors was collected from maternal questionnaires. Educational level was categorized into low, medium and high. Pregnancy and infant birth characteristics were obtained from the Finnish Medical Birth Register kept by the Finnish Institute for Health and Welfare (www.thl.fi). Participating parents gave written informed consent on behalf of the expected child. All DNA samples and data were pseudonymized. The Ethics Committee of the Hospital District of Southwest Finland approved the study protocol and all methods were carried out in accordance with Helsinki declaration.

### Study population and the outcome of RRIs

**Figure 1:**
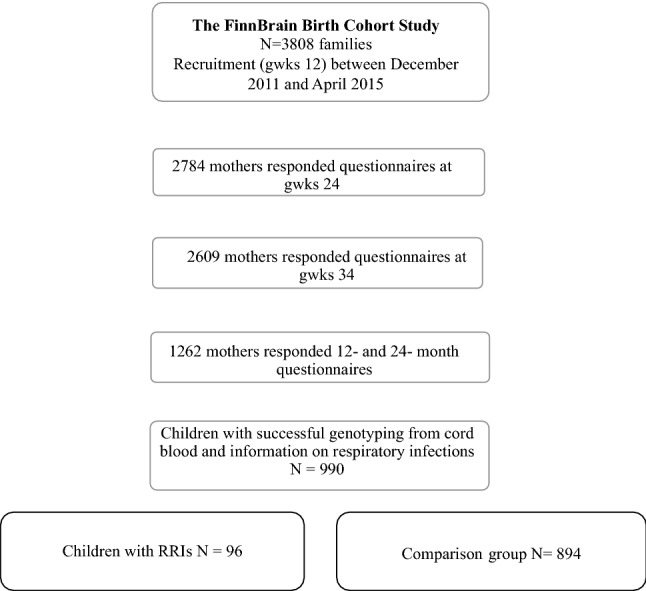
Flowchart of study population. *gwk* gestational week, *RRIs* recurrent respiratory infections.

The current study population was based on the Cohort participants who had responded to the questionnaires on child health at the child ages of 12 and 24 months (n = 1262 children). Of these, 990 (78.4%) children provided a cord blood sample with successful genotyping and, thus, composed the study population for the current analyses. The study outcome was presence of RRIs at the child age of 12 or/and 24 months. Children with RRIs (n = 96; 9.7% of 990 subjects) were identified by maternal reports on a question “Has your child had recurrent infections” (yes/no) at the child age of 12 and 24 months. Answer “yes” to this single question at either age, based on our previous report^16^, determined the RRI group. The remaining study population with successful genotyping from cord blood (n = 894; 90.3% of 990 subjects) were regarded as the comparison group (Fig. 1). Physician visits for RTIs, physician-given diagnoses (RTI, rhinitis, cough, acute otitis media, bronchiolitis, or pneumonia), and antibiotic treatments for RTIs were inquired monthly with questionnaires.

### Genetic analyses

Cord blood was used for genotyping. DNA samples were extracted according to standard procedures at the Finnish Institute for Health and Welfare. DNA samples were genotyped with Illumina Infinium PsychArray BeadChip (Illumina, San Diego, CA) comprising 603132 SNPs at Estonian Genome Centre, Tartu, Estonia and quality control was performed with PLINK 1.9 (www.cog-genomics.org/plink/1.9/)^19^. Markers were removed for missingness (> 5%) and Hardy–Weinberg equilibrium (*P* < 1 × 10^–6^). Individuals were checked for missing genotypes (> 5%), relatedness (identical by descent calculation, PI_HAT > 0.2) and population stratification (multidimensional scaling). Genotyped data was imputed with IMPUTE2^20^ using the 1000 Genomes project phase 3 haplotypes and a haplotype set of 1941 whole genome sequenced Finnish individuals as reference panels. In all, 16 variants previously reported to be associated with function of innate immune system were selected for this study were rs1333969 and rs273259 at *IFI44L*^21,22^, rs1990760, rs35667974, and rs3747517 at *IFIH1*^23^, rs2243250 at *IL4*^24^, rs1800795 at *IL6*^9^, rs1800896 at *IL10*^24^, rs2275913 at *IL17A*^25^, rs5030737, rs1800450, and rs1800451 at *MBL2*^26^, rs4986790 at *TLR4*^27^, rs5743708 at *TLR2*^28^*,* and rs361525 and rs1800629 at *TNF*^9^*.* In *MBL2* gene, the major allele was labelled as “A” and the three variant alleles “D” in rs5030737, “B” in rs1800450, and “C” in rs1800451 were collectively labelled as "O", since heterozygosity at any of these SNPs (A/O) results in reduced serum MBL concentration and homozygosity or combination of the minor alleles (O/O) results in almost absent serum MBL^26^.

### Prenatal psychological distress: maternal symptoms of depression and anxiety during gestation

Mothers’ psychological distress was evaluated by self-reports at gwks 34. The Edinburgh Postnatal Depression Scale (EPDS) was used to measure symptoms of depression. The EPDS consists of 10 questions scored on a 4-point Likert scale (0–3 points/item)^29^. The total scores range between 0 and 30. The EPDS has proven to be a valid, reliable and effective screening tool for identifying patients at risk for perinatal depression^30^. The Symptom Checklist-90 (SCL-90)^31^, anxiety subscale was used to measure symptoms of anxiety and Pregnancy-Related Anxiety Questionnaire, revised 2 (PRAQ-R2)^32^ to measure pregnancy-specific symptoms of anxiety. The anxiety subscale consists of 10 items scored on a 5-point Likert scale (0–4 points/item), and the range of the total sum score is 0–40. The items of PRAQ-R2 can be divided into three subscales: Fear of Giving Birth (Factor 1), Worries about Bearing a Physically or Mentally Handicapped Child (Factor 2), and Concern about Own Appearance (Factor 3). Scores on each item ranged from 1 to 5 and total sum scores were calculated.

### Statistical analyses

Sociodemographic and other background data and mother-reported symptom scores in prenatal testing of psychological distress were compared between the RRI and comparison groups by using the t-test, χ^2^, or Mann–Whitney U test, when appropriate. Firstly, the association between preselected genetic variants of innate immune system and RRI was tested with the logistic regression analysis implemented with PLINK^19^ and adjusted for sex. The alleles were classified according to allele frequency so that the associations depend additively on the minor alleles (Additive model)–that is, children having two minor alleles (minor/minor) were labelled as (2), minor/major genotype as (1), and major/major genotype as (0), respectively.

Secondly, the interactions between genotype and maternal prenatal psychological distress symptoms were analyzed using logistic regression models with the form:

$$ Risk \, for \, RRIs \, = \, Distress \, + \, Genotype \, + \, Distress \, \times \, Genotype, $$

where *RRI* was a binary variable, *Distress* was categorized dichotomously either with EPDS (< 10 points, low; ≥ 10 points, high) or PRAQ-R2 (< 26 points, low; ≥ 26 points, high), and *Genotype* was a continuous variable labeled as above. We used this dichotomized distress symptom category (low *vs.* high distress) for our genotype–environment models as there is evidence that in healthy general populations also low levels of maternal prenatal distress may potentially be harmful to offspring^33^. Scoring ≥ 10 points in the EPDS questionnaire was regarded as high distress since this represents possible clinical depression^23^. With the PRAQ-R2 the highest quartile of scores (≥ 26 points) was regarded as high distress. The additive model was also used to analyze the associations between genotypes and RRIs separately in children with prenatal exposure to maternal psychological distress and in non-exposed children. Finally, the analyses were done post hoc separately for males and females in exploratory manner to test the hypothesis of sex difference. All statistical analyses were conducted using IBM SPSS 24.0 (SPSS Inc, Chicago, IL) and R v. 3.6.1. (R Core Team, 2019, https://www.R-project.org/).

## Results

### Study population characteristics

Of the RRI group, 27 of 96 (28%) responded “yes” to the question “Has your child had recurrent infections” at 12 months, 56 (58%) at 24 months, and 13 (14%) at both ages of the child. The proportions of children with documented outcomes that reflect high disease burden from RTIs were clearly higher in the RRI group defined by mother’s assessment than in the comparison group. Thirty-one percent of children with RRIs had more than five respiratory infections before the age of 1 year whereas in the comparison group 4% of the children had more than five RTIs. Children in RRI group had significantly more antibiotic treatments before 2 years of age and 42% of the children had tympanostomy tubes inserted. Population characteristics are detailed in Table 1. As previously shown in our cohort, RRI status was associated with maternal prenatal symptoms of depression (EPDS total sum score, *P* = 0.009) and pregnancy-specific anxiety (PRAQ-R2 total sum score, *P* = 0.028), but not significantly to general anxiety symptoms (Table 1)^16^.

**Table 1:** Study population characteristics in children with recurrent respiratory tract infections (RRIs) and the comparison group.

	RRI group n = 96	Comparison group n = 894	*P*
Mothers’ age, years, mean (SD)	31 (4.2)	30 (4.6)	0.15
**Maternal education (%)**^**a**^			
Low (up to 12 years)	25.5	36.0	0.02
Medium (13–15 years)	44.0	30.0	
High (over 15 years)	30.5	34.0	
Maternal smoking (%)^b^ (yes/no)	10.0	13.0	0.34
Child sex male (%)	58	52	0.21
Gestational weeks, mean (SD)	39.7 (1.5)	39.8 (1.4)	0.28
Apgar score at 1 min, mean (SD)	8.6 (1.4)	8.6 (1.3)	0.99
Tympanostomy tube insertions (%)^c^	42	1	< 0.001
**Antibiotic treatments before 1 year of age (%)**			< 0.001
0	34	77	
1–4	45	22	
5–10	21	1	
> 10	0	0	
**Antibiotic treatments before 2 years of age (%)**^**d**^			< 0.001
0	0	25	
1–4	31	63	
5–10	67	12	
> 10	2	0	
**Respiratory tract infections before 1 year of age (%)**			< 0.001
0	27	68	
1–4	42	28	
5–10	30	4	
> 10	1	0	
**Respiratory tract infections before 2 years of age (%)**^**e**^			< 0.001
0	0	38	
1–4	21	42	
5–10	60	18	
> 10	19	2	
**Prenatal maternal psychological distress questionnaires, total scores at gwks 34**^**f**^**, mean (IQR)**			
EPDS	5.32 (2–8.4)	4.5 (3–6.7)	0.009
PRAQ	24.0 (18–28)	22.5 (17–27)	0.028
SCL-90	3.7 (0–5)	3.3 (0–4)	0.085

*EPDS* the Edinburgh Postnatal Depression Scale, *gwks* gestational weeks, *PRAQ-R2* the Pregnancy-Related Anxiety Questionnaire–Revised2, *SCL-90* the Symptom Checklist 90, anxiety subscale.

*P* values are based on χ^2^ for categorical and t-test and Mann–Whitney U test for continuous variables indicating the group comparisons. Values are presented as numbers (percentages), means (standard deviation, SD) or medians (interquartile range, IQR).

^a^RRI-group n = 94, comparison group n = 851.

^b^RRI-group n = 93, comparison group n = 894.

^c^RRI-group n = 67, comparison group = 262.

^d^RRI-group n = 49, comparison group n = 176.

^e^RRI-group n = 48, comparison group n = 94.

^f^RRI-group n = 93, comparison group n = 816.

### Associations between innate immune gene variants and RRIs

*IL6* (rs1800795) minor allele G was associated with elevated risk for RRIs (adjusted odds ratio [aOR], 1.55; 95% confidence interval [CI], 1.14–2.12; *P* = 0.006, adjusted for sex) (Table 2). The most common forward strand allele was C in the comparison group and G in the RRI group. The IL-6 genotype distribution in the comparison group was G/G (20%), G/C (50%), and C/C (30%) whereas in the RRI group it was G/G (27%), G/C (56%), and C/C (16%). Other studied SNPs of innate immune genes were not significantly associated with elevated or decreased risk for RRIs. However, two polymorphisms of the interferon pathway participating gene *IFI44L*, rs1333969 (aOR 0.71; 95% CI 0.50–1.01; *P* = 0.058) and rs273259 (aOR 0.74; 95% CI 0.53–1.02; *P* = 0.070) appeared as possibly protective for RRIs. For the variant forms of *MBL2,* aOR for RRIs was 1.28 (95% CI 0.83–1.97).

**Table 2:** The association between minor alleles at studied single nucleotide polymorphisms of innate immune genes and recurrent respiratory infections.

Gene	SNP	MAF	N	OR (95% CI)	*P*
*IFI44L*	rs1333969	0.27	990	0.71 (0.49–1.01)	0.058
*IFI44L*	rs273259	0.36	982	0.74 (0.53–1.02)	0.070
*IFIH1*	rs1990760	0.41	990	1.03 (0.77–1.39)	0.83
*IFIH1*	rs35667974	0.02	990	1.01 (0.31–3.27	0.99
*IFIH1*	rs3747517	0.31	990	0.82 (0.59–1.14)	0.24
*IL4*	rs2243250	0.32	980	0.92 (0.67–1.27)	0.62
*IL6*	rs1800795	0.46	976	1.55 (1.14–2.12)	0.006
*IL10*	rs1800896	0.45	990	1.00 (0.75–1.35)	0.98
*IL17A*	rs2275913	0.44	898	0.98 (0.72–1.33)	0.88
*MBL2*	rs5030737	0.06	990	1.28 (0.83–1.97)^**a**^	0.26
*MBL2*	rs1800450	0.13	990		
*MBL2*	rs1800451	0.01	990		
*TLR4*	rs4986790	0.10	990	0.83 (0.49–1.41)	0.49
*TLR2*	rs5743708	0.03	957	1.18 (0.49–2.84)	0.72
*TNF*	rs361525	0.02	990	1.18 (0.45–3.08)	0.73
*TNF*	rs1800629	0.15	990	0.75 (0.47–1.18)	0.21

*N* number of genotyped children, *CI* confidence interval, *MAF* minor allele frequency, *OR* odds ratio for recurrent respiratory infections, *SNP* single nucleotide polymorphism.

Tested with the logistic regression analysis implemented with PLINK (Purcell) and adjusted for sex.

^a^Calculated for minor allele at any of the three *MBL2* SNPs.

### Interaction between gene variants and maternal prenatal psychological distress

For the GxE (maternal prenatal psychological distress) analyses, *IL6* rs1800795 and *IFI44L* rs1333969 and rs273259 polymorphisms were selected based on their associations with RRIs (*IL6* variants predisposing and *IFI44L* variants protecting, Table 2). No significant interactions were identified for the risk for RRIs for these gene variants and the dichotomized symptom score measures of the EPDS and PRAQ-R2 questionnaires reflecting maternal distress at gwks 34 in all children or separately in males or females (Table 3). Analyses were further adjusted for maternal EPDS score at 1 year of age, this did not alter the results.

**Table 3:** The interaction of the exposure to prenatal maternal psychological distress and genotype for the risk of recurrent respiratory infections in all children and separately in males and females.

Psychological distress at gwks 34—genotype^a^	N	OR	95% CI	*P*
**EPDS scores high (≥ 10 points)-IL6 (rs1800795)**				
All	893	1.24	0.56–2.73	0.60
Male	469	1.40	0.43–4.53	0.58
Female	423	1.07	0.36–3.22	0.90
**PRAQR2 scores high (≥ 26 points)-IL6 (rs1800795)**				
All	894	1.15	0.61–2.16	0.67
Male	469	1.68	0.73–3.85	0.22
Female	424	0.67	0.25–1.8	0.43
**EPDS scores high (≥ 10 points)-IFI44L (rs1333969)**				
All	905	1.15	0.42–3.11	0.78
Male	475	1.55	0.44–5.44	0.49
Female	429	0.73	0.13–3.96	0.71
**PRAQR2 scores high (≥ 26 points)-IFI44L (rs1333969)**				
All	906	1.62	0.77–3.42	0.20
Male	475	1.44	0.55–3.73	0.46
Female	430	1.77	0.54–5.83	0.34
**EPDS scores high (≥ 10 points)-IFI44L (rs273259)**				
All	904	0.62	0.24–1.61	0.32
Male	474	1.01	0.32–3.20	0.99
Female	429	0.25	0.04–1.41	0.12
**PRAQR2 scores high (≥ 26 points)-IFI44L (rs273259)**				
All	905	1.31	0.67–2.54	0.43
Male	474	1.21	0.52–2.77	0.66
Female	430	1.26	0.41–3.83	0.69

*CI* confidence interval, *EPDS* the Edinburgh Postnatal Depression Scale, *OR* odds ratio, *PRAQ-R2* the Pregnancy-Related Anxiety Questionnaire–Revised2.

Studied as interaction logistic regression models and *P* values for the interaction terms.

^a^Additive Model: major/major allele = 0, minor/major allele = 1 and minor/minor allele = 2.

Finally, we performed post hoc analyses in exploratory manner to investigate the association between *IL6* (rs1800795) variant and RRIs separately for males and females and stratified according to the level of prenatal exposure to maternal distress. In males carrying the minor G allele and born to mothers with highest quartile of scores in the PRAQ-R2 questionnaire, the risk for RRIs was elevated (OR 1.96; 95% CI 1.04–3.67; *P* = 0.04), whereas no genetic association was found in males born to mothers with low scores in PRAQ-R2 (Table 4). On the contrary, in females the risk for RRIs associated with *IL6* G allele was independent on the maternal prenatal stress (Table 4).

**Table 4:** The association of *IL6* (rs1800795)^a^ variant genotype and the risk for RRIs in groups stratified according to prenatal maternal psychological distress exposure, assessed separately for males and females.

	N	OR	95% CI	*P*
**Males**				
PRAQR2 scores low (< 26 points, the reference category)	317	1.17	0.68–2.01	0.58
PRAQR2 scores high (≥ 26 points)	152	1.96	1.04–3.67	0.04
EPDS scores low (< 10 points, the reference category)	394	1.40	0.90–2.16	0.13
EPDS scores high (≥ 10 points)	75	1.95	0.65–5.81	0.23
**Females**				
PRAQR2 scores low (< 26 points, the reference category)	310	1.83	0.97–3.44	0.06
PRAQR2 scores high (≥ 26 points)	114	1.23	0.58–2.61	0.59
EPDS scores low (< 10 points, the reference category)	354	1.57	0.90–2.75	0.11
EPDS scores high (≥ 10 points)	69	1.69	0.66–4.34	0.28

Odds ratio (OR) for recurrent respiratory infections, Interleukin 6 (*IL6*).

^a^Additive Model: major/major allele = 0, minor/major allele = 1 ja minor/minor allele = 2.

## Discussion

Of the preselected set of candidate gene variants previously related to impaired function of innate immunity in early childhood, we found an association between *IL6* (rs1800795) gene variant and risk for RRIs during the first 2 years of life. As we had reported earlier, mothers of children with RRIs had higher levels of psychological distress during gestation^16^. There was no significant interaction between maternal stress and *IL6* or *IFI44L* gene variants with regard to risk for RRIs. However, our exploratory analyses stratified by child’s sex were suggestive of sex-dependent genotype–environment-related vulnerability for RRIs.

The studied polymorphism *IL6* (rs1800795) is located in the promoter region of the *IL6* gene and has been shown to affect the transcriptional regulation and plasma levels of the IL-6 cytokine^34^. In previous studies, both the G and C alleles have been associated with stronger IL-6 responses, but recent studies have demonstrated that the C allele is associated with lower plasma levels of IL-6^34^. In our study, the majority of children in RRI group had the G-allele, suggesting that stronger IL-6 responses are associated with RRIs. The cytokine levels are related to symptoms of RTIs, and it has been suggested that children who are high acute-phase cytokine producers because of various cytokine polymorphisms may be more susceptible to recurrent otitis media^10^. These findings highlight the complexity of the relations between genetic variations, innate immune responses, and recurrence of RTIs.

Both *IFI44L* polymorphisms (rs1333969 and rs273259) affect interferon pathway activation and have role in the development of RTI symptoms. In our study, children with variant forms of *IFI44L* had lower risk for RRIs, although the difference was not significant. The studied SNPs in genes *IL10, IFIH1, MBL2, IL17A, TLR4*, *TLR2*, *IL4,* and *TNF* have been previously related to altered immune responses, recurrent RTIs, recurrent AOMs, or nasopharyngeal bacterial colonization in young children^5–12,35–37^, but we did not find significant associations between these genetic variants and susceptibility to RRIs. Reasons for this may include differences in size and age range of the study populations and in definition of outcome between our study and earlier studies.

In our cohort, mothers of children in the RRI group had more symptoms of pre-and postnatal depression and anxiety than mothers in the comparison group^16^. Mother’s high plasma cortisol levels during pregnancy due to psychological distress expose the fetus to high cortisol levels^38^. This in turn can cause structural and functional reorganization of physiological systems such as hypothalamic–pituitary–adrenal axis and immune system in the offspring^39,40^. IL-6 release is mediated by glucocorticoids^41^. Also IL-6 has been shown to stimulate the adrenocortical axis, and thus, the secretion of IL-6 and glucocorticoids form a bi-directional loop^41^ Adults with psychological stress have more symptomatic upper respiratory tract infections and have also been found to have elevated IL-6 levels^42^. Beijers and collegues showed that maternal prenatal anxiety and maternal cortisol levels were both associated with increased number of child RTIs during the first year of life^43^. Pregnancy-specific anxiety has been found to be a relevant predictor of child health outcomes^44^. It may be that PRAQ scores refer to a mother’s adjustment to pregnancy and reveal an important aspect of the prenatal stress exposure. Of note, PRAQ scores are often normally distributed in the general population and even high scores do not refer to a defined illness or disorder.

Our interaction analysis suggested that maternal prenatal stress and the *IL6* gene variant are not linked to each other. However, performing the exploratory analyses separately for males and females, revealed that in males the *IL6* rs1800795 G allele increased the risk for RRIs only in association with maternal pregnancy-specific anxiety. In females, such association was not observed. Child’s sex contributes to physiological and anatomical differences influencing the innate and adaptive immune functions^45,46^. It has been previously demonstrated that male and female offspring respond to prenatal stress in a different way^47,48^. Female fetuses are possibly more sensitive to milder forms of stress whereas male fetuses seem to require more intense exposure during the prenatal period in order to develop alterations in the hypothalamic–pituitary–adrenal axis function^49^. Our exploratory analyses suggest that a genotype–environment effect between *IL6* gene variant and prenatal maternal distress may present in a sexually dimorphic manner.

### Strengths and limitations

Main strengths of this study are prospective study design based on general population and the measurement of maternal psychosocial distress with validated questionnaires. This study population was retrieved from the general population-based birth cohort and therefore the group of mothers with high PD symptom levels was small and only a low number of mothers scored above the clinical thresholds of depressive (EPDS) or anxiety (SCL) symptoms. The study sample of 990 families presents about 25% of the FinnBrain Birth Cohort of 3808 subjects (Fig. 1, Flowchart of study population). As previously shown in our cohort, mothers with more severe symptoms of depression and anxiety tend to drop out. For example, mothers who responded to the gwks 34 questionnaires, were older, more often primiparous, had higher socioeconomic status, smoked less frequently and reported lower depressive symptom scores at gwks 14 compared to those who did not return the third trimester questionnaires^18^. It can be suggested that the association between maternal prenatal stress and a child´s RRIs would have been even stronger in the whole cohort because of higher proportion of symptomatic mothers compared to our study sample.

Definition of RRI group was based on maternal report, which may have resulted in certain inaccuracy in characterization of the phenotype. However, the maternal report reflects importance of child’s RRIs for the family. Another limitation is that the findings on potential sex differences are based on post hoc analyses stratified by sex in exploratory manner, thus not formally testing the sex differences but rather providing material for the hypotheses of future research. The number of these subpopulations was also quite small, indicating that this finding needs to be interpreted with caution and replicated by further research performed in larger study populations.

## Conclusions

The strongest evidence for the association between the susceptibility to RRIs and genetic variants of the innate immune system was observed for *IL6* polymorphism. Overall, there was no interaction between prenatal maternal psychological distress and child genotype. Nevertheless, prenatal maternal pregnancy-specific anxiety suggested a sex-dependent GxE on the risk of RRIs. Future research on biological susceptibility to infections should take into account potential sex differences and prenatal exposure to stress as an environmental risk factor.

## Abbreviations

AOM: Acute otitis media
EPDS: The Edinburgh postnatal depression scale
Gwk: Gestational week
IFIH1: Interferon induced with helicase C domain 1
IFI44L: Interferon induced protein 44 like
IL: Interleukin
MBL: Mannose-binding lectin
PRAQ-R2: The Pregnancy-Related Anxiety Questionnaire-Revised 2
RRI: Recurrent respiratory infections
RTI: Respiratory tract infection
SCL-90: The symptom checklist-90
SNP: Single nucleotide polymorphism
TLR: Toll-like receptor
TNF: Tumor necrosis factor
